# iThermo: A Sequence-Based Model for Identifying Thermophilic Proteins Using a Multi-Feature Fusion Strategy

**DOI:** 10.3389/fmicb.2022.790063

**Published:** 2022-02-22

**Authors:** Zahoor Ahmed, Hasan Zulfiqar, Abdullah Aman Khan, Ijaz Gul, Fu-Ying Dao, Zhao-Yue Zhang, Xiao-Long Yu, Lixia Tang

**Affiliations:** ^1^School of Life Sciences and Technology, Center for Informational Biology, University of Electronic Science and Technology of China, Chengdu, China; ^2^School of Computer Science and Engineering, University of Electronic Science and Technology of China, Chengdu, China; ^3^Sichuan Artificial Intelligence Research Institute, Yibin, China; ^4^Tsinghua Shenzhen International Graduate School, Institute of Biopharmaceutical and Health Engineering, Tsinghua University, Shenzhen, China; ^5^School of Materials Science and Engineering, Hainan University, Haikou, China

**Keywords:** thermophilic proteins, protein feature extraction, feature selection, neural network, iThermo

## Abstract

Thermophilic proteins have important application value in biotechnology and industrial processes. The correct identification of thermophilic proteins provides important information for the application of these proteins in engineering. The identification method of thermophilic proteins based on biochemistry is laborious, time-consuming, and high cost. Therefore, there is an urgent need for a fast and accurate method to identify thermophilic proteins. Considering this urgency, we constructed a reliable benchmark dataset containing 1,368 thermophilic and 1,443 non-thermophilic proteins. A multi-layer perceptron (MLP) model based on a multi-feature fusion strategy was proposed to discriminate thermophilic proteins from non-thermophilic proteins. On independent data set, the proposed model could achieve an accuracy of 96.26%, which demonstrates that the model has a good application prospect. In order to use the model conveniently, a user-friendly software package called iThermo was established and can be freely accessed at http://lin-group.cn/server/iThermo/index.html. The high accuracy of the model and the practicability of the developed software package indicate that this study can accelerate the discovery and engineering application of thermally stable proteins.

## Introduction

In the field of industrial and biotechnology development, researchers usually increase the temperature to shorten the enzymatic reaction time (Tang et al., 2017). However, the increase in temperature leads to the denaturation of protein, resulting in the loss of protein activity. Maintaining the activity of protein under increasing temperature conditions is a hot topic in the current engineering field. It is well known that temperature is crucial to cellular life. It has been reported that some organisms can live in a high-temperature environment. In general, the organisms that survive at an optimal growth temperature (OGT) below 50°C are regarded as mesophilic organisms, and the organisms that can survive at the OGT of 50°C or above are called thermophilic organisms (Gromiha and Suresh, 2008). Thermophiles can produce thermally stable proteins and even effectively resist high temperatures of up to 120°C (Fan et al., 2016; Tang et al., 2017). Therefore, the study of proteins produced by thermophilic organisms is significant for the development of enzyme engineering (Huang and Gong, 2020; Wang et al., 2020; Alim et al., 2021; Suresh et al., 2021; Zou et al., 2021).

There have been many studies on thermophilic proteins. It is found that the thermal stability of proteins is related to amino acid distribution in proteins (Fukuchi and Nishikawa, 2001; Zhou et al., 2008). In addition to amino acid distribution, dipeptide composition (DC) contributes effectively to protein thermal stability (Ding et al., 2004; Zhang and Fang, 2007; Nakariyakul et al., 2012). In addition, previous studies have reported that the factors affecting the thermal stability of proteins also include hydrophobicity (Saraboji et al., 2005; Miyazaki et al., 2006; Gromiha et al., 2013), hydrogen bonding (Bleicher et al., 2011), residues and inter-residue contacts (Gromiha, 2001; Meruelo et al., 2012), helical polar surfaces (Jayaraman et al., 2006), side-chain interactions (Kumar et al., 2000), and salt bridges (Sadeghi et al., 2006; Ge et al., 2008).

Based on these characteristics, some computational models have been developed to predict thermophilic proteins (Wang et al., 2020). Gromiha and Suresh (2008) developed a neural network-based model. They reported 89 and 91% accuracy using 5-fold cross-validation and independent dataset, respectively. Lin and Chen (2011) built the most reliable benchmark dataset at that time, including 915 thermophilic proteins and 793 non-thermophilic proteins. Using amino acid composition (AAC) and dipeptide composition as inputs of support vector machine (SVM), the accuracy for thermophilic proteins and non-thermophilic proteins was 93.8 and 92.7%, respectively. Then, the genetic algorithm combined with SVM was applied to the prediction problem (Wang et al., 2011; Lv et al., 2020c). Nakariyakul et al. (2012) established a computational model on the same dataset constructed by Lin and Chen (2011). Their model achieved an accuracy of 93.3% in jackknife cross-validation. In recent years, combined with AAC, evolutionary information, and acid dissociation constant, Fan et al. (2016) built a prediction model with an accuracy of 93.5%. Tang et al. (2017) proposed a two-steps discrimination method using the same dataset and achieved an accuracy of 94.44% in 5-fold cross-validation. A voting algorithm for thermophilic proteins prediction has achieved an accuracy of 93.03% (Li J. et al., 2019). Feng et al. (2020) developed a reduced AAC-based model and obtained an accuracy of 98.2%. Guo et al. (2020) used the feature dimension reduction technique to identify thermophilic protein and reported an accuracy of 96.02%.

Although much work has been done to predict thermophilic proteins, the availability of a reliable benchmark dataset, the development of an accurate model based on multi-feature fusion, and the construction of a software package still need to be further improved. Therefore, this study constructed the most reliable benchmark dataset. Subsequently, an accurate model was developed based on this dataset. Based on the model, a software package was established. The following sections will introduce these processes in detail.

## Materials and Methods

**FIGURE 1:**
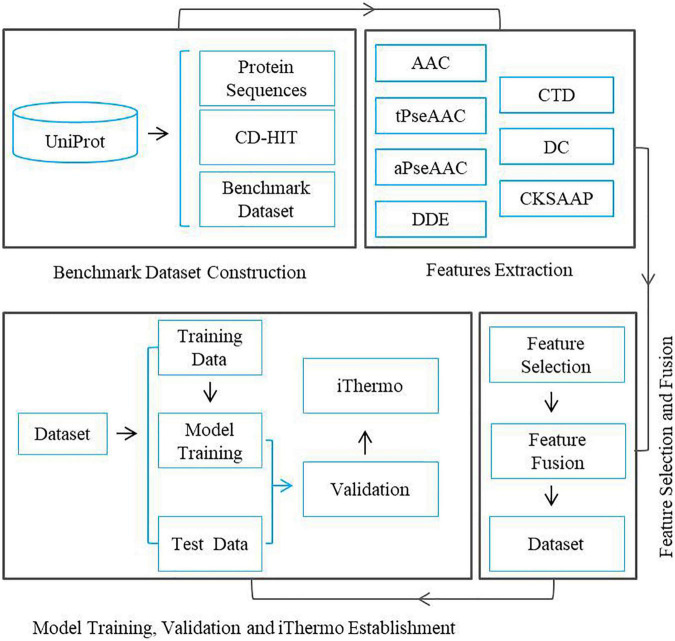
Flow chart of a framework for predicting thermophilic proteins.

The fundamental framework of the present research work includes the following five steps: (1) benchmark dataset construction, (2) feature extraction, (3) feature selection, (4) feature fusion, (5) model training, and (6) software package establishment. The flow chart of the framework is illustrated in Figure 1.

### Dataset

The cornerstone of a robust and reliable model is to generate a reliable and strict benchmark dataset. In previous literature, scholars used 50°C as a cutoff to construct a benchmark dataset. However, this criterion did not seem objective because proteins might be stable even above the OGT of microorganisms. For instance, a protein produced by microorganisms living at 45°C is likely not to denature at 60°C. According to the 50°C cutoff criterion, this protein is included in the negative dataset, but it should be included in the positive dataset as it is still stable above the 50°C. To eradicate this effect as much as possible, we used Lin and Chen’s (2011) strict and objective standard to generate a benchmark dataset. According to Lin and Chen’s (2011) criterion, the proteins in the microorganism with OGT > 60°C and <30°C were regarded as thermophilic and non-thermophilic proteins, respectively. Of course, even after using Lin and Chen’s (2011) criterion, the effect mentioned still exists but not as strongly as when compared to the 50°C cutoff criterion. All protein sequences were extracted from a universal protein resource (UniProt). Subsequently, the following steps were used to ensure the quality of protein data: (I) the proteins which have been manually reviewed remained; (II) proteins containing ambiguous residues were excluded; (III) sequences which are a fragment of other proteins were excluded; (IV) proteins which infer from prediction or homology were excluded; (V) to remove redundancy and homology bias, CD-HIT program (Huang et al., 2010) was used by setting a cutoff of sequence identity to 30%. As a result, the final benchmark dataset contained 1,443 non-thermophilic and 1,366 thermophilic proteins. Our final dataset contains only a few thousand proteins because the growth temperature of some microorganisms is known (Li G. et al., 2019) and UniProt contains few confirmed proteins. We only included experimental data. Moreover, noise and redundancy were removed, which also caused a reduction in the number of proteins. For training model, the dataset was divided into 80:20 ratios; model was trained on 80% dataset and validated on 20% dataset.

### Feature Extraction

Protein sequences were transformed into numerical vectors to identify thermophilic proteins by machine learning methods (Liu et al., 2019, 2020; Li et al., 2021; Zhang et al., 2021a,b). To accomplish this task, we used the iFeature program (Chen et al., 2018) to generate seven kinds of protein features, namely amino acid composition (AAC), traditional pseudo amino acid composition (tPseAAC), amphiphilic pseudo amino acid composition (aPseAAC), the composition of *k*-spaced amino acid pairs (CKSAAP), dipeptide composition (DC), dipeptide deviation from the expected mean (DDE), and composition, transition, and distribution (CTD). These features will be described in detail in the following sections.

#### Amino Acid Composition

Amino acid composition (Bhasin and Raghava, 2004; Lv Z. et al., 2021) refers to the occurrence frequencies of 20 amino acid residues in a protein sequence and is defined as:


(1) $$f\,\left(t\right)=\frac{N\,\left(t\right)}{N},t\in \left\{A,C,D,\ldots ,Y\right\}$$

where *f(t)* represents the frequency of *t* amino acid, *N*(*t*) indicates the total number of *t* amino acids in a protein sequence of length *N*.

#### Traditional Pseudo Amino Acid Composition

Traditional pseudo amino acid composition was used to describe residues correlation based on their physicochemical properties (Chou, 2001). The descriptor uses the 20+λ dimensional vectors to represent the protein sequence. The 20 and λ dimensions denote the amino acid composition and sequence correlation factor, respectively.

For any protein *P*, its tPseAAC can be represented as:


(2) $$P={\left[A_{1},A_{2},A_{3},\ldots ,A_{20},A_{20+1},\ldots ,A_{20+\lambda }\right]}^{T}$$

where the 20+λ dimension elements can be formulated as:


(3) $$p_{u}=\left\{ \begin{array}{c} \frac{f_{u}}{\sum_{\mu -1}^{20}f_{u}+\omega \,\sum_{k-1}^{\lambda }\tau_{k}},1\leq \mu \leq 20\\ \frac{\omega \,\tau_{u-20}}{\sum_{\mu -1}^{20}f_{u}+\omega \,\sum_{k-1}^{\lambda }\tau_{k}},21\leq \mu \leq 20+\lambda \end{array}\right.$$

where *P*_*u*_ and *w* denote the feature vector and weight factor, respectively. Here, we set *w* to 0.05 for saving computational time. The *f*_*u*_ shows the amino acids occurrence frequency in a protein *P*. *τ_*k*_* represents the *k*-tire sequence correlation factor which is given below by formula:


(4) $$\tau_{k}=\frac{1}{L-k}\sum_{i=1}^{L-k}J_{i,i+k},\left(k<L\right)$$

(5) $$J_{i,i+k}=\frac{1}{3}\{{\left[H_{1}\left(R_{i}\right)-H_{1}\left(R_{i+k}\right)\right]}^{2}+{\left[H_{2}\left(R_{i}\right)-H_{2}\left(R_{i+k}\right)\right]}^{2}+{\left[M\left(R_{i}\right)-M\left(R_{i+k}\right)\right]}^{2}\}$$

where *H*_1_(*R*_*i*_) is the hydrophobicity value, *H*_2_(*R*_*i*_) is the hydrophilicity value, and *M* (*R*_*i*_) is the side chain mass of the amino acid residue *R*_*i*_. For detailed descriptions about tPseAAC, please refer to the literature (Chou, 2001).

#### Amphiphilic Pseudo Amino Acid Composition

This descriptor incorporates a partial sequence-order effect to the amino acids based on hydrophobicity and hydrophilicity (Chou, 2005). According to aPseAAC, a protein is represented as follows:


(6) $$P=\left[A_{1},A_{2},A_{3},\ldots ,A_{20},A_{20+1},\ldots ,A_{20+\lambda },\ldots ,A_{20+2\,\lambda }\right]$$

where the first 20-dimension elements represent the AAC, and the remaining dimensions represent the sequence correlation factor similar to tPseAAC. For further details about aPseAAC, please refer to the literature (Chou, 2005).

#### Composition of *k*-Spaced Amino Acid Pairs

The CKSAAP describes the frequencies of paired amino acids separated by any amino acid with the symbol *k*. The value of *k* may vary from 0 to 5 (Chen et al., 2007). CKSAAP for (*k* = 0) was formulated as:


(7) $$F_{0}={\left(\frac{F_{\mathrm{AA}}}{N_{0}},\frac{F_{\mathrm{AC}}}{N_{0}},\frac{F_{\mathrm{AD}}}{N_{0}},\ldots ,\frac{F_{\mathrm{YY}}}{N_{0}}\right)}_{400}$$

where *F*_0_ represents the CKSAAP for (*k* = 0), *F* represents the frequency of zero spaced paired amino acids, and *N*_0_ represents total zero spaced amino acid pairs.

#### Dipeptide Composition

Dipeptide composition is the frequencies of dipeptides in a protein sequence and is defined as:


(8) $$D_{c}\,\left(g,h\right)=\frac{N\,\left(g,h\right)}{N-1}$$

where *Dc*(*g,h*) denotes the frequency of dipeptide (*g,h*), while *N*(*g,h*) denotes the number of times dipeptide (*g*,*h*) present in the protein sequence containing total dipeptides *N* (Saravanan and Gautham, 2015).

#### Dipeptide Deviation From Expected Means

Dipeptide deviation from expected means proposed by Saravanan and Gautham (2015), involves the combination of dipeptide composition (DC), theoretical mean (*T*_*m*_), and theoretical variance (*T*_*v*_), which was defined as:


(9) $$\mathrm{DDE}\,\left(g,h\right)=\frac{D_{c}\,\left(g,h\right)-T_{m}\,\left(g,h\right)}{\sqrt{T_{v}\,\left(g,h\right)}}$$

where,


(10) $$T_{m}\,\left(g,h\right)=\frac{C_{g}}{C_{N}}\times \frac{C_{h}}{C_{N}}$$

where *Cg* indicates the total codons code for amino acid *g*, and *Ch* indicates the total codons code for amino acid *h*. *CN* is the number of codons except for the stop codons.

The theoretical variance *Tv* is defined as:


(11) $$T_{v}\,\left(g,h\right)=\frac{T_{m}\,\left(g,h\right)\,\left(1-T_{m}\,\left(g,h\right)\right)}{N-1}$$

where *N* denotes the length of the sequence.

#### Composition, Transition, and Distribution

According to the characteristics of amino acids, 20 amino acids can be categorized as polar, neutral, and hydrophobic. According to the definition of CTD, composition (C) is the percent occurrence of polar, neutral, and hydrophobic residues; transition (T) indicates the frequency in transition; and distribution (D) is the position of the first 25, 50, 75, and 100% amino acid of each group.


(12) $$C\,\left(r\right)=\frac{N\,\left(r\right)}{N},r\in \left\{p\,o\,l\,a\,r,n\,e\,u\,t\,r\,a\,l,h\,y\,d\,r\,o\,p\,h\,o\,b\,i\,c\right\}$$

where *N*(*r*) and *N* indicate the number of amino acids of type *r* and sequence length, respectively (Tomii and Kanehisa, 1996; Dubchak et al., 1999).

### Feature Selection

Redundant features and noise affect the prediction performance of the model. In order to get the best prediction performance, it is necessary to remove redundant features and noise using feature selection methods (Tang et al., 2020; Zhang Z. M. et al., 2020; Dao et al., 2021c). In this study, the analysis of variance (ANOVA; Tang et al., 2018) was applied for feature ranking, and a sequential backward selection strategy was used to pick out optimal features. The following section will introduce the method briefly.

Analysis of variance (ANOVA) can be used to select the best feature subsets based on *F*-value. *F*-value is the ratio of the variance between the sample types and the variance within the samples. A feature’s greater *F*-value implies that the feature can contribute more to discriminating between positive and negative samples.

*F*-value for a feature *m* can be calculated as:


(13) F⁢(m)=s2b⁢(m)s2w⁢(m)

where *s^2^_*b*_* is the variance between the features and *s^2^_*w*_* is the variance with each feature’s sample. These variances can be represented as:


(14) s2b⁢(m)=∑i=1Kni⁢(∑j=1nifi⁢j⁢(m)ni-∑i=1K∑j=1nifi⁢j⁢(m)∑i=1Kn=i)2⁢/⁢d⁢fb

(15) s2w⁢(m)=∑i=1K∑j=1ni(fi⁢j⁢(m)-∑i=1K∑j=1nifi⁢j⁢(m)∑i=1Kni)2⁢/⁢d⁢fw

where *K* denotes the total features, *N* denotes the total samples, *fij(m)* denotes the *m*-th feature of the *j*-th sample in the *i*-th group, and *n*_*i*_ denotes sample in the *i*-th group. The degree of freedom for between features *df*_*b*_ and within features *df*_*w*_ was *K*-1 and *N*-1, respectively. Detailed descriptions about ANOVA can be referred to as reference (Tang et al., 2018).

### Classification

For classification, we examined a number of classifiers, including Support Vector Machine (SVM; Tang et al., 2017), K Nearest Neighbor (KNN; Zuo et al., 2013; Zulfiqar et al., 2021a), Random Forest (RF), and Multi-layer Perceptron (MLP) for training the model. The following sections will introduce these classifiers briefly.

#### Support Vector Machine

Support vector machine maps the features in multi-dimensional space and defines the optimal hyperplane to separate the two classes using a kernel function. Different kernels functions can be used in SVM. Because of the non-linearity of data, we used radial basis function (RBF), which can be represented for vectors *a* and *b* by formula as:


(16) $$K\,\left(a,b\right)=\exp\,\left(-\gamma \,{||a-b||}^{2}\right)$$

where γ denotes the training parameter. The tradeoff between a lower training error and large margins is controlled by a regularization factor *C*. In the present study, the value of γ and *C* was set to 0.0001 and 900, respectively. For further details about SVM, see (Joachims, 1998).

#### Random Forest

Random forest is based on ensemble methodology to predict the final results. It involves various decision trees, each containing a decision node, leaf node, and root node. A leaf node is the output of each decision tree. The final output is based on the majority voting system (Lv et al., 2020a). If we have attributes Θ of a vector *x* and decision tree based on these attributes is *h*(*x*, Θ), then the random forest can be defined as:


(17) $$f=\left\{h\,\left(x,\Theta_{k}\right)\right\},k=1,2,\ldots ,k$$

In the present study, the hyperparameters maximum depth, minimum sample split, and n_estimators were set 100, 10, and 500, respectively. For a detailed algorithm of random forest, refer to reference (Breiman, 2001).

#### K Nearest Neighbors

K nearest neighbor is the most commonly used classifier. It represents the feature vectors as points in feature space and calculates the distance between these points. The final decision is made based on the distance between these points. KNN commonly uses the Euclidean distance as the distance metric.

The Euclidean distance is given below:


(18) $$\mathrm{dist}\,\left(M,N\right)=\sqrt{\sum_{i=1}^{n}c_{i}\,{\left(m_{i}-n_{i}\right)}^{2}}$$

where *M* and *N* are two feature vectors while *m* shows feature space dimensionality (Uddin et al., 2019). The present study applied the KNN classifier using hyper parameters n-neighbor, *P*, and leaf-size as 6, 1, and 2, respectively.

#### Multi-Layer Perceptron

Deep learning is also a popular method in bioinformatics (Dao et al., 2021a,b; Lv H. et al., 2021; Wang et al., 2021; Zulfiqar et al., 2022). MLP is a feed-forward neural network containing input, hidden, and output layers for receiving input data, processing data, and performing final prediction, respectively. It trains the network using a supervised learning technique known as backpropagation. The following equation describes the output result of each trained neuron.


(19) $$f\,\left(\alpha \right)=f\,\left(\sum_{i=1}w_{i}\,x_{i}+b\right)$$

where *x*_*i*_ indicates the input values of the firing neuron, *w*_*i*_ are their weights, *f* represents the activation function, and *b* presents the activation threshold of the neuron. For a detailed MLP algorithm, refer to the reference (Taud and Mas, 2018). In the present study, rectified linear activation unit (ReLU) was used as an activation function in the hidden layer; for the outer layer activation function, a sigmoid was used. Input, hidden, and output layers containing 83, 100, and 1 neuron, respectively, were used to train the model. The detail of hyperparameters is presented in Table 1.

**TABLE 1:** Best hyperparameters for MLP classifier.

Hyperparameters	Value
Batch size	60
Epochs	1200
Learning rate	0.001
Momentum	0.8
Decay	1e^–8^
Nesterov	True
Verbose	1

### Performance Evaluation

In order to evaluate the overall model performance, the following parameters were used (Lv et al., 2020b,d; Shao et al., 2021).


(20) $$\mathrm{Sn}=\frac{\mathrm{TP}}{\mathrm{TP}+\mathrm{FN}}$$

(21) $$\mathrm{Sp}=\frac{\mathrm{TN}}{\mathrm{TN}+\mathrm{FP}}$$

(22) $$\mathrm{Acc}=\frac{\mathrm{TP}+\mathrm{TN}}{\mathrm{TP}+\mathrm{FP}+\mathrm{TN}+\mathrm{FN}}$$

(23) $$\mathrm{MCC}=\frac{\mathrm{TP}\times \mathrm{TN}-\mathrm{FP}\times \mathrm{FN}}{\sqrt{\left(T\,P+F\,N\right)\,\left(T\,N+F\,N\right)\,\left(T\,P+F\,P\right)\,\left(T\,N+F\,P\right)}}$$

where *Sn, Sp, Acc*, and *MCC* denote sensitivity, specificity, accuracy, and Matthews’s correlation coefficient. Thermophilic proteins classified as thermophilic were denoted *TP* (true positive), Non-thermophilic proteins classified as non-thermophilic were denoted *TN* (true negative), Non-thermophilic proteins classified as thermophilic were denoted by *FP* (false positive), and thermophilic proteins classified as non-thermophilic were denoted by *FN* (false negative).

## Results and Discussion

### Performance Evaluation

For performance evaluation, seven descriptors including AAC, tPseAAC, aPseAAC, DC, DDE, CKSAAP, and CTD were used to create numerical vectors from protein sequences. In order to use these numerical vectors, MLP-based models were trained to evaluate their performances. Results showed that the AUC are 0.9723, 0.9551, 0.9519, 0.8812, 0.9081, 0.9081, and 0.9786 for AAC, tPseAAC, aPseAAC, DC, DDE, CKSAAP, and CTD, respectively (as shown in Table 2). In order to remove the redundant features and improve the prediction performance of the model, a feature selection method should be used to pick out the optimal features from each descriptor. In this work, ANOVA was used to rank features for selecting the best feature subsets from the seven types of descriptors. Table 2 also recorded the performance of each descriptor after feature selection. It showed that AAC, tPseAAC, aPseAAC, DC, DDE, CKSAAP, and CTD produced the best AUC of 0.9735, 0.9580, 0.9610, 0.9143, 0.9165, 0.8349, and 0.9644, respectively. Obviously, the performance of each descriptor increased after the feature selection except the CTD descriptor; therefore, we considered all features of CTD in our study.

**TABLE 2:** Performance of descriptors before and after feature selection and in feature fusion.

	Descriptors	SN	SP	AAC	MCC	AUC
Before feature selection	ACC	0.9304	0.9308	0.9306	0.8626	0.9723
	tPseAAC	0.9011	0.8793	0.8899	0.7914	0.9551
	aPseAAC	0.8901	0.8720	0.8808	0.7714	0.9519
	DC	0.7546	0.8720	0.8149	0.5963	0.8812
	DDE	0.8022	0.8374	0.8203	0.6319	0.9081
	CKSAAP	0.7912	0.5398	0.6619	0.3855	0.7365
	CTD	0.9377	0.9100	0.9235	0.8612	0.9786
After feature selection	ACC	0.9524	0.9239	0.9377	0.8902	0.9735
	tPseAAC	0.8938	0.8962	0.8950	0.7943	0.9580
	aPseAAC	0.8971	0.8824	0.8895	0.7863	0.9610
	DC	0.8859	0.8754	0.8416	0.6620	0.9143
	DDE	0.7802	0.8651	0.8238	0.6430	0.9165
	CKSAAP	0.7070	0.8374	0.7740	0.5156	0.8349
	CTD	0.9167	0.9135	0.9150	0.8330	0.9644
	Feature fusion	0.9634	0.9619	0.9626	0.9269	0.9864

The above results and analysis have demonstrated that each descriptor has useful information to discriminate thermophilic proteins from non-thermophilic proteins. We adopted a feature fusion strategy to include the valuable information of all selected features from each descriptor in model training. In feature fusion, the selected optimal feature subsets of seven descriptors were fused and inputted into the MLP classifier to distinguish thermophilic proteins from non-thermophilic proteins. Table 2 shows that the AUC increased to 0.9864, suggesting that feature fusion is very effective and has made an outstanding contribution to improving the model’s prediction performance.

### Performance Comparison on Different Algorithms

**FIGURE 2:**
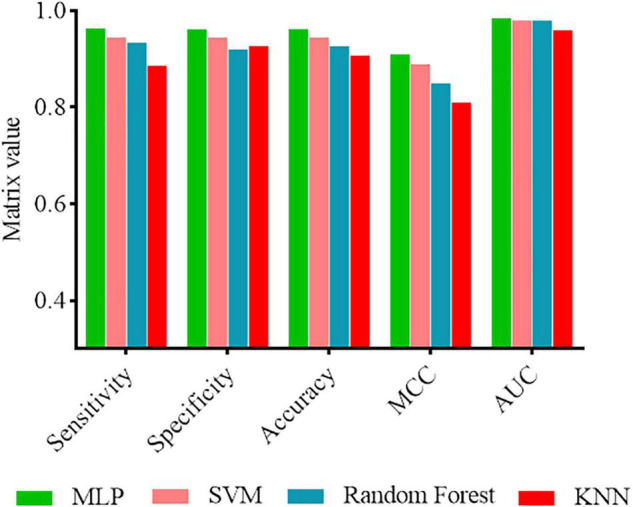
Performance comparison of MLP classifier with other classifiers.

In order to demonstrate that the MLP classifier has better prediction performance, we also investigated the performance of other machine learning methods, including SVM, Random forest, and KNN. These methods were trained and tested using the same fused features. The results are recorded in Figure 2. As shown in Figure 2, the performance of MLP classifiers was better than other classifiers. Therefore, we considered using a MLP-based model to establish a software package.

### Comparison to Other Models

Many models have been proposed for thermophilic protein identification (Gromiha and Suresh, 2008; Lin and Chen, 2011; Wang et al., 2011; Nakariyakul et al., 2012; Fan et al., 2016; Tang et al., 2017; Li J. et al., 2019; Feng et al., 2020; Guo et al., 2020). All proposed models were established based on machine learning methods and were evaluated by cross-validation. However, our model was examined on independent data. Moreover, the benchmark dataset used in the present study was rigorous and objective. Moreover, most of these published works did not establish available tools that are not only non-practical but also prevent us from making a fair comparison. The only available web-server for the identification of thermophilic proteins was established by Lin and Chen (2011). We performed a comparison with the web server using the same validation dataset. Their model (Lin and Chen, 2011) displayed 95.30% accuracy, while our model produced an accuracy of 96.26%.

### Feature Analysis

Our model produces good prediction performance and shows that the characteristics used can effectively characterize thermophilic proteins. Thus, we performed an analysis on features based on their contribution to model performance. In order to find feature contribution, we used permutation feature importance. The contribution of features to the performance of the model is represented in Supplementary Table 1. The following section will analyze the feature of each descriptor briefly.

The composition and arrangement of amino acids determine the unique function of a protein. At present, the research on thermophilic proteins uses the composition characteristics of amino acids. The current study involves a detailed analysis of AAC. We found that the frequencies of alanine (A), lysine (K), valine (V), isoleucine (I), glutamine (Q), aspartic acid (D), tyrosine (Y), serine (S), glutamic acid (E), and threonine (T) were significantly different between the two classes. It is speculated that these amino acids have crucial information in providing either thermophilicity or non-thermophilicity to proteins. Tyrosine contributed the most to model performance among these amino acids and showed the weight 0.0249 ± 0.0080. Moreover, lysine, glutamic acid, glutamine, and aspartic acid also contributed well to model performance and showed the weights 0.0033 ± 0.0026, 0.0041 ± 0.0017, 0.0036 ± 0.0049, and 0.0162 ± 0.0036, respectively. Glutamate, lysine, tyrosine, glutamic acid, and aspartic acid residues were more common in thermophilic proteins than non-thermophilic proteins. Thermophilic proteins contain highly charged amino acids, which contribute to the thermal stability of proteins. Lysine, glutamine, aspartic acid, and glutamic acid residues belong to charged amino acids, while tyrosine belongs to polar amino acids. These amino acids participate in forming salt bridges and hydrogen bonds, which provide thermal stability to proteins. These results are consistent with previous studies (Liu et al., 2011; Wang et al., 2011; Panja et al., 2020).

Valine and isoleucine showed good ability for thermophilic protein identification. In permutation feature importance, valine and isoleucine showed the weights 0.0201 ± 0.0056 and 0.0101 ± 0.0028, respectively. Isoleucine and valine are hydrophobic amino acids. It has been reported that hydrophobicity contributes to the thermal stability of proteins, as during protein folding, hydrophobic amino acids get buried inside the protein to form a hydrophobic core; this hydrophobic core contributes to the thermal stability of proteins (Baldwin, 2007; Gromiha and Suresh, 2008).

**FIGURE 3:**
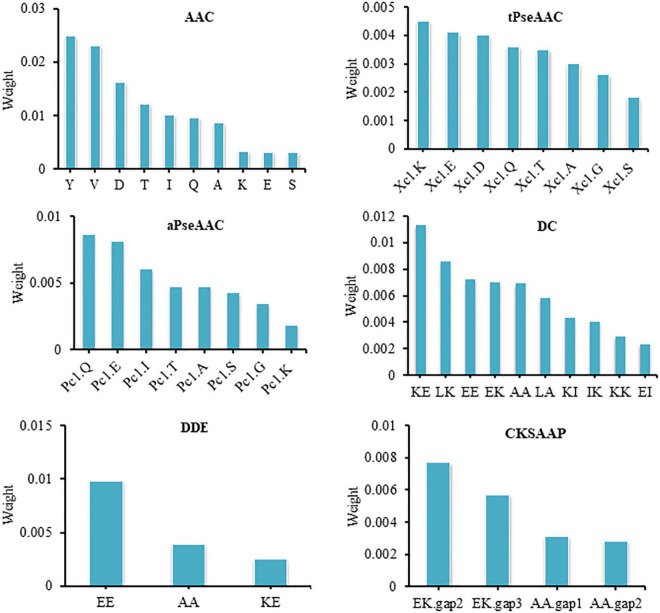
Contribution of features of all descriptors to model performance.

Amino acid alanine, threonine, and serine indicated an important role in model performance and showed the weights 0.0087 ± 0.0018, 0.0122 ± 0.0045, and 0.0031 ± 0.0039, respectively. Figure 3 illustrates the contribution of AAC features to model performance. Non-thermophilic proteins contain more alanine, threonine, and serine residues than thermophilic proteins, consistent with a previous study by Cambillau and Claverie (2000). Alanine carries less charge, while threonine and serine are neutral amino acids, so these amino acids are rarely involved in forming hydrogen bonds and salt bridges, indicating that the proteins enriched with these amino acids can be prone to thermal denaturation (Lin and Chen, 2011).

Amino acid composition is an excellent descriptor to discriminate thermophilic proteins from non-thermophilic proteins. Previous studies have also confirmed the contribution of AAC to protein classification tasks (Gromiha and Suresh, 2008; Mahmoudi et al., 2016). Although AAC plays a good role in protein classification, it also lacks sequence information. The traditional tPseAAC and aPseAAC (Chou, 2001, 2005) are good options for the lack of sequence information in AAC. Wang et al. (2011) and Chen et al. (2016) also confirmed the critical role of these descriptors in protein classification.

Both tPseAAC and aPseAAC are used to describe the sequence information of amino acid residues in protein sequence. In tPseAAC, Xc1.K, Xc1.E, Xc1.D, Xc1.Q, Xc1.T, Xc1.A, Xc1.G, and Xc1.S were valuable features with the weights of 0.0045 ± 0.0019, 0.0041 ± 0.0017, 0.0040 ± 0.0029, 0.0036 ± 0.0049, 0.0035 ± 0.0015, 0.0030 ± 0.0029, 0.0026 ± 0.0027, and 0.0018 ± 0.0026, respectively (Figure 3). The features Pc1.Q, Pc1.E, Pc1.I, Pc1.T, Pc1.A, Pc1.S, Pc1.G, and Pc1.K in aPseAAC presented important contribution to model performance. They showed the weights 0.0086 ± 0.0030, 0.0081 ± 0.0046, 0.0060 ± 0.0057, 0.0047 ± 0.0030, 0.0047 ± 0.0026, 0.0042 ± 0.0030, 0.0034 ± 0.0023, and 0.0018 ± 0.0014, respectively (Figure 3). Our in-depth analysis showed that hydrophobic amino acid and polar amino acid based features were more frequent in thermophilic protein, while uncharged and neutral amino acid based features were more frequent in non-thermophilic proteins.

Dipeptides are also an important feature to distinguish thermophilic proteins from non-thermophilic proteins. Our statistical analysis showed that the occurrence frequencies of KE, LK, EE, EK, AA, LA, KI, IK, KK, and EI have a considerable variance between the two classes of proteins. The ranking of features also confirmed the role of these dipeptides in model performance. Dipeptide KE, LK, EE, EK, AA, LA, KI, IK, KK, and EI showed the weights 0.0113 ± 0.0029, 0.0086 ± 0.0045, 0.0072 ± 0.0019, 0.0070 ± 0.0040, 0.0069 ± 0.0043, 0.0058 ± 0.0010, 0.0043 ± 0.0013, 0.0040 ± 0.0017, 0.0029 ± 0.0026, and 0.0023 ± 0.0017, respectively (Figure 3). Dipeptide KE, LK, EE, EK, KI, IK, KK, and EI have charged at biological pH, showing a great trend of forming salt bridges and hydrogen bonds, which contributes to the thermal stability of proteins. AA and LA have poor charge capability and were found more in non-thermophilic proteins (Nakariyakul et al., 2012; Panja et al., 2020). Previous studies have also confirmed the role of dipeptide composition in identifying thermophilic proteins (Gromiha et al., 2005; Lin and Chen, 2011). MLP model trained on these selected features reveals that these features have good capability to distinguish thermophilic proteins.

The dipeptide deviation from the expected mean also showed meaningful information for the identification of thermophilic proteins. Features including EE, AA, and KE deviation from expected mean showed good ability to identify thermophilic proteins (Table 2). The dipeptide deviation for EE, AA, and KE showed the weights 0.0098 ± 0.0032, 0.0039 ± 0.0028, and 0.0025 ± 0.0012, respectively (Figure 3). Previous studies have also reported the effective contribution of dipeptide deviating from the expected mean in protein classification tasks (Saravanan and Gautham, 2015; Ho Thanh Lam et al., 2020). In addition to these dipeptide-related descriptors, we also considered the composition of *k*-spaced amino acid pairs, representing the paired amino acid frequency separated by any other amino acid. It is a valuable descriptor and has been widely used in previous studies for protein classification (Jang et al., 2020; Ju and Wang, 2020; Zhang L. et al., 2020; Zulfiqar et al., 2021b). In the present study, E^∗∗^K, E^∗∗∗^K, A^∗∗^A, and A^∗^A were found to be containing meaningful information for thermophilic protein identification and showed the weight 0.0077 ± 0.0041, 0.0057 ± 0.0014, 0.0031 ± 0.0034, and 0.0028 ± 0.0015, respectively (Figure 3).

Composition, transition, and distribution involves the composition, transition, and distribution of hydrophobic, polar, and neutral residues. Like other descriptors, the hydrophobic and polar residue-based features of CTD were more frequent in thermophilic proteins while neutral residues-based features were more frequent in non-thermophilic proteins. Permutation feature importance of descriptor CTD is represented in Supplementary Table 2. In previous studies, the CTD has been extensively used for protein classification purposes. Wang et al. (2011) and Zulfiqar et al. (2021b) also reported CTD as a valuable descriptor for thermophilic protein identification. In the present study, the CTD showed an excellent capability to identify thermophilic proteins (Table 2). For CTD, all features performed better than the selected features, so we used CTD features without selection. MLP model trained on CTD features performed good results (Table 2).

## iThermo

In addition to proposing a validated model, it is essential to establish a tool to promote the application of the model. To meet this requirement, we established an application software package, iThermo http://lin-group.cn/server/iThermo/index.html. The software package can provide easy access to the model. The software package can be used to make efficient and accurate predictions for thermophilic proteins. It is anticipated that this study will provide a good alternative to laborious, expensive, and time-consuming laboratory practices.

## Conclusion

Thermophilic proteins can withstand the harsh conditions of elevated temperature. Thermophilic proteins have attracted much attention in biotechnology and industrial applications. High temperature leads to protein denaturation, so it is urgent to establish a reliable identification method of thermophilic proteins. The identification of thermophilic proteins based on biochemistry is time-consuming, laborious, and expensive. The computational method-based thermophilic protein identification can provide an attractive choice for rapid, cost-effective, and straightforward identification of thermophilic proteins.

Considering this urgency, this study constructed a reliable benchmark dataset and used this dataset to train an MLP classifier. The model has good performance on an independent dataset and can accurately identify thermophilic proteins with an accuracy of 96.20%. In order to facilitate access to the model, a software package was also established. The high performance of the model and its availability as flexible packaging can provide a good choice for thermophilic protein study.

## Data Availability Statement

The original contributions presented in the study are included in the article/Supplementary Material, further inquiries can be directed to the corresponding authors.

## Author Contributions

LT, X-LY, and Z-YZ conceived and designed the study. ZA conducted the experiments, implemented algorithms, performed the analysis, and wrote the manuscript. ZA, AK, and F-YD established a software package. IG and HZ reviewed and edited the manuscript. LT supervised the study. All authors contributed to the article and approved the submitted version.

## Conflict of Interest

The authors declare that the research was conducted in the absence of any commercial or financial relationships that could be construed as a potential conflict of interest.

## Publisher’s Note

All claims expressed in this article are solely those of the authors and do not necessarily represent those of their affiliated organizations, or those of the publisher, the editors and the reviewers. Any product that may be evaluated in this article, or claim that may be made by its manufacturer, is not guaranteed or endorsed by the publisher.
