# Supplementary material for: iThermo: A Sequence-Based Model for Identifying Thermophilic Proteins Using a Multi-Feature Fusion Strategy
Source: Front Microbiol. 2022 Feb 22;13:790063. doi: 10.3389/fmicb.2022.790063 (PMC8902591; doi:10.3389/fmicb.2022.790063)
Supplement: Supplementary file 1 [file Data_Sheet_1.docx]

iThermo: a sequence-based model for identifying thermophilic protein using multi feature fusion strategy

# Supplementary Figures and Tables

## Supplementary Tables

**Table 1: Contribution of features in model performance**

(Features of descriptor DDE are represented in lower case to differentiate them from DC features)

| **S.No.** | **Feature** | **Weight** |
| --- | --- | --- |
| 1 | Y | 0.0249 ± 0.0080 |
| 2 | secondarystruct.G2 | 0.0231 ± 0.0058 |
| 3 | V | 0.0201 ± 0.0056 |
| 4 | D | 0.0162 ± 0.0036 |
| 5 | hydrophobicity_CASG920101.G3 | 0.0159 ± 0.0082 |
| 6 | hydrophobicity_ARGP820101.G3 | 0.0158 ± 0.0066 |
| 7 | hydrophobicity_ZIMJ680101.G2 | 0.0136 ± 0.0039 |
| 8 | hydrophobicity_PONP930101.G2 | 0.0122 ± 0.0031 |
| 9 | T | 0.0122 ± 0.0045 |
| 10 | KE | 0.0113 ± 0.0029 |
| 11 | hydrophobicity_ZIMJ680101.G3 | 0.0111 ± 0.0025 |
| 12 | charge.G1 | 0.0103 ± 0.0025 |
| 13 | I | 0.0101 ± 0.0028 |
| 14 | ee | 0.0098 ± 0.0032 |
| 15 | Q | 0.0096 ± 0.0026 |
| 16 | polarity.G1 | 0.0095 ± 0.0038 |
| 17 | charge.G2 | 0.0093 ± 0.0032 |
| 18 | A | 0.0087 ± 0.0018 |
| 19 | Pc1.Q | 0.0086 ± 0.0030 |
| 20 | LK | 0.0086 ± 0.0045 |
| 21 | Pc1.E | 0.0081 ± 0.0046 |
| 22 | EK.gap2 | 0.0077 ± 0.0041 |
| 23 | solventaccess.G1 | 0.0076 ± 0.0011 |
| 24 | hydrophobicity_CASG920101.G1 | 0.0076 ± 0.0048 |
| 25 | EE | 0.0072 ± 0.0019 |
| 26 | hydrophobicity_FASG890101.G2 | 0.0070 ± 0.0023 |
| 27 | hydrophobicity_FASG890101.G3 | 0.0070 ± 0.0032 |
| 28 | EK | 0.0070 ± 0.0040 |
| 29 | normwaalsvolume.G2 | 0.0069 ± 0.0037 |
| 30 | AA | 0.0069 ± 0.0043 |
| 31 | Pc1.I | 0.0060 ± 0.0057 |
| 32 | hydrophobicity_ENGD860101.G2 | 0.0059 ± 0.0029 |
| 33 | LA | 0.0058 ± 0.0010 |
| 34 | EK.gap3 | 0.0057 ± 0.0014 |
| 35 | secondarystruct.G1 | 0.0051 ± 0.0012 |
| 36 | Pc1.T | 0.0047 ± 0.0030 |
| 37 | Pc1.A | 0.0047 ± 0.0026 |
| 38 | polarity.G3 | 0.0045 ± 0.0009 |
| 39 | Xc1.K | 0.0045 ± 0.0019 |
| 40 | polarity.G2 | 0.0045 ± 0.0020 |
| 41 | hydrophobicity_PRAM900101.G1 | 0.0045 ± 0.0016 |
| 42 | hydrophobicity_CASG920101.G2 | 0.0044 ± 0.0038 |
| 43 | normwaalsvolume.G3 | 0.0044 ± 0.0029 |
| 44 | hydrophobicity_ENGD860101.G3 | 0.0043 ± 0.0014 |
| 45 | KI | 0.0043 ± 0.0013 |
| 46 | Pc1.S | 0.0042 ± 0.0030 |
| 47 | hydrophobicity_FASG890101.G1 | 0.0042 ± 0.0021 |
| 48 | Xc1.E | 0.0041 ± 0.0017 |
| 49 | hydrophobicity_ARGP820101.G1 | 0.0041 ± 0.0010 |
| 50 | Xc1.D | 0.0040 ± 0.0029 |
| 51 | IK | 0.0040 ± 0.0017 |
| 52 | hydrophobicity_PONP930101.G1 | 0.0040 ± 0.0022 |
| 53 | aa | 0.0039 ± 0.0028 |
| 54 | secondarystruct.G3 | 0.0039 ± 0.0033 |
| 55 | polarizability.G3 | 0.0037 ± 0.0018 |
| 56 | Xc1.Q | 0.0036 ± 0.0049 |
| 57 | charge.G3 | 0.0036 ± 0.0027 |
| 58 | normwaalsvolume.G1 | 0.0035 ± 0.0019 |
| 59 | Xc1.T | 0.0035 ± 0.0015 |
| 60 | Pc1.G | 0.0034 ± 0.0023 |
| 61 | solventaccess.G3 | 0.0034 ± 0.0009 |
| 62 | polarizability.G1 | 0.0034 ± 0.0031 |
| 63 | K | 0.0033 ± 0.0026 |
| 64 | solventaccess.G2 | 0.0033 ± 0.0039 |
| 65 | hydrophobicity_ARGP820101.G2 | 0.0031 ± 0.0015 |
| 66 | hydrophobicity_PONP930101.G3 | 0.0031 ± 0.0027 |
| 67 | E | 0.0031 ± 0.0018 |
| 68 | S | 0.0031 ± 0.0039 |
| 69 | AA.gap1 | 0.0031 ± 0.0034 |
| 70 | polarizability.G2 | 0.0030 ± 0.0015 |
| 71 | Xc1.A | 0.0030 ± 0.0029 |
| 72 | KK | 0.0029 ± 0.0026 |
| 73 | hydrophobicity_PRAM900101.G3 | 0.0028 ± 0.0026 |
| 74 | AA.gap2 | 0.0028 ± 0.0015 |
| 75 | hydrophobicity_ENGD860101.G1 | 0.0028 ± 0.0025 |
| 76 | Xc1.G | 0.0026 ± 0.0027 |
| 77 | ke | 0.0025 ± 0.0012 |
| 78 | EI | 0.0023 ± 0.0017 |
| 79 | Pc1.K | 0.0018 ± 0.0014 |
| 80 | Xc1.S | 0.0018 ± 0.0026 |
| 81 | hydrophobicity_PRAM900101.G2 | 0.0016 ± 0.0036 |
| 82 | hydrophobicity_ZIMJ680101.G1 | 0.0014 ± 0.0024 |

**Table 2: Contribution of CTD features in model performence**

| **S.No.** | **Feature** | **Weight** |
| --- | --- | --- |
| 1 | secondarystruct.G2 | 0.0231 ± 0.0058 |
| 2 | hydrophobicity_CASG920101.G3 | 0.0159 ± 0.0082 |
| 3 | hydrophobicity_ARGP820101.G3 | 0.0158 ± 0.0066 |
| 4 | hydrophobicity_ZIMJ680101.G2 | 0.0136 ± 0.0039 |
| 5 | hydrophobicity_PONP930101.G2 | 0.0122 ± 0.0031 |
| 6 | hydrophobicity_ZIMJ680101.G3 | 0.0111 ± 0.0025 |
| 7 | charge.G1 | 0.0103 ± 0.0025 |
| 8 | polarity.G1 | 0.0095 ± 0.0038 |
| 9 | charge.G2 | 0.0093 ± 0.0032 |
| 10 | solventaccess.G1 | 0.0076 ± 0.0011 |
| 11 | hydrophobicity_CASG920101.G1 | 0.0076 ± 0.0048 |
| 12 | hydrophobicity_FASG890101.G2 | 0.0070 ± 0.0023 |
| 13 | hydrophobicity_FASG890101.G3 | 0.0070 ± 0.0032 |
| 14 | normwaalsvolume.G2 | 0.0069 ± 0.0037 |
| 15 | hydrophobicity_ENGD860101.G2 | 0.0059 ± 0.0029 |
| 16 | secondarystruct.G1 | 0.0051 ± 0.0012 |
| 17 | polarity.G3 | 0.0045 ± 0.0009 |
| 18 | polarity.G2 | 0.0045 ± 0.0020 |
| 19 | hydrophobicity_PRAM900101.G1 | 0.0045 ± 0.0016 |
| 20 | hydrophobicity_CASG920101.G2 | 0.0044 ± 0.0038 |
| 21 | normwaalsvolume.G3 | 0.0044 ± 0.0029 |
| 22 | hydrophobicity_ENGD860101.G3 | 0.0043 ± 0.0014 |
| 23 | hydrophobicity_FASG890101.G1 | 0.0042 ± 0.0021 |
| 24 | hydrophobicity_ARGP820101.G1 | 0.0041 ± 0.0010 |
| 25 | hydrophobicity_PONP930101.G1 | 0.0040 ± 0.0022 |
| 26 | secondarystruct.G3 | 0.0039 ± 0.0033 |
| 27 | polarizability.G3 | 0.0037 ± 0.0018 |
| 28 | charge.G3 | 0.0036 ± 0.0027 |
| 29 | normwaalsvolume.G1 | 0.0035 ± 0.0019 |
| 30 | solventaccess.G3 | 0.0034 ± 0.0009 |
| 31 | polarizability.G1 | 0.0034 ± 0.0031 |
| 32 | solventaccess.G2 | 0.0033 ± 0.0039 |
| 33 | hydrophobicity_ARGP820101.G2 | 0.0031 ± 0.0015 |
| 34 | hydrophobicity_PONP930101.G3 | 0.0031 ± 0.0027 |
| 35 | polarizability.G2 | 0.0030 ± 0.0015 |
| 36 | hydrophobicity_PRAM900101.G3 | 0.0028 ± 0.0026 |
| 37 | hydrophobicity_ENGD860101.G1 | 0.0028 ± 0.0025 |
| 38 | hydrophobicity_PRAM900101.G2 | 0.0016 ± 0.0036 |
| 39 | hydrophobicity_ZIMJ680101.G1 | 0.0014 ± 0.0024 |
